# New Signal Functions to Measure the Ability of Health Facilities to Provide Routine and Emergency Newborn Care

**DOI:** 10.1371/journal.pmed.1001340

**Published:** 2012-11-13

**Authors:** Sabine Gabrysch, Giulia Civitelli, Karen M. Edmond, Matthews Mathai, Moazzam Ali, Zulfiqar A. Bhutta, Oona M. R. Campbell

**Affiliations:** 1 University of Heidelberg, Institute of Public Health, Heidelberg, Germany; 2 Sapienza University of Rome, Public Health and Infectious Diseases Department, Rome, Italy; 3 University of Western Australia, School of Paediatrics and Child Health, Subiaco, Australia; 4 Department of Maternal, Newborn, Child & Adolescent Health, World Health Organization, Geneva, Switzerland; 5 Department of Reproductive Health and Research, World Health Organization, Geneva, Switzerland; 6 Division of Women and Child Health, The Aga Khan University, Karachi, Pakistan; 7 London School of Hygiene & Tropical Medicine, Faculty of Epidemiology and Population Health, London, United Kingdom

## Abstract

Based upon an expert survey and consensus method, Sabine Gabrysch and colleagues recommend new signal functions to monitor and track facilities' provision of routine and emergency newborn care.

**Summary Points**

- Emergency obstetric care (EmOC) signal functions, reflecting health facilities' capacity to respond to important obstetric complications, are widely used to construct indicators of service provision.
- However, no signal functions are agreed for emergency newborn care (EmNC), except newborn resuscitation, or for routine non-emergency care for mothers and newborns.
- Current large-scale facility survey efforts mainly collect data on the established EmOC functions, and two EmNC functions (newborn resuscitation and prevention of mother to child transmission of HIV). Routine maternal or newborn care data are not regularly included.
- We propose maternal and newborn signal functions, focussing on delivery and postnatal care, that could be used to characterize both routine and emergency care in health facilities.

## Measuring Obstetric and Newborn Functionality of Health Facilities

Skilled delivery care providers working in the right environment can prevent, detect, and treat complications, thus contributing to a reduction in stillbirths and maternal and neonatal mortality. Skilled care is most often approximated by assessing whether women deliver with a midwife, doctor, or other skilled birth attendant [1], but ensuring an enabling environment for uncomplicated (routine) and complicated childbirth is an equally important requirement for skilled care [2].

Emergency obstetric care (EmOC) signal functions, a shortlist of key life-saving obstetric interventions, have been used to assess the functionality of health facilities with respect to EmOC and to construct indicators of service provision. They are thought to reflect responsiveness of the health services to the main obstetric complications at basic and comprehensive level, roughly corresponding to health centre level (with midwives) and first-referral hospital level (with physicians). The availability and density of facilities capable of providing EmOC have been suggested as useful health system output indicators for monitoring supply-side progress towards having sufficient services for reducing maternal mortality [3]–[6].

In 1986 the World Health Organization (WHO) published *Essential Obstetric Functions at First Referral Level* [7], which describes key obstetric functions that hospitals should provide. These functions focussed on emergency treatment for complications (surgical obstetrics, anaesthesia, blood replacement, manual procedures) but also included a monitoring function (the partograph), a preventive function (family planning support, which prevents pregnancy rather than complications in pregnant women), and an emergency newborn function (neonatal resuscitation). EmOC signal functions, described in the 1997 UN guidelines [3], focussed more sharply on eight signal functions for treating the five main causes of maternal mortality (haemorrhage, hypertensive diseases of pregnancy, sepsis, obstructed labour, and unsafe abortion), and removed the monitoring, prevention, and neonatal treatment signal functions (partograph, surgical contraception, and newborn resuscitation). EmOC was subdivided into basic and comprehensive EmOC, with the former comprising six medical functions (parenteral antibiotics, parenteral oxytocic drugs, parenteral anticonvulsants, manual removal of placenta, removal of retained products, and assisted vaginal delivery) and the latter adding surgical capability (Caesarean section) and blood transfusion.

There have been attempts to develop signal functions for children [8], but no similar signal functions have been widely agreed for newborns, except for newborn resuscitation. The latter function had been part of the 1986 Essential Obstetric Functions, was removed from EmOC as defined in 1997, and then reintroduced as a basic EmOC signal function in the 2009 UN handbook [4]. Similarly, there has been little focus on signal functions for routine care for mothers or newborns; routine care saves lives by preventing complications or by intervening early before life-threatening complications develop [9]. For example, effective elements of routine care which should be provided to all women and all newborns include using a partograph to detect prolonged labour in time, ensuring a clean delivery, providing active management of the third stage of labour (AMTSL) to reduce the risk of post-partum haemorrhage, and encouraging early breastfeeding and keeping the baby warm.

In view of the emerging consensus on the continuum of care linking mother and child, and the links between routine care and care for complications, this paper argues for expanding our assessment of health facility capability from EmOC signal functions alone, to a broader range of capabilities including emergency newborn care (EmNC) and routine care for mothers and newborns. While family planning, antenatal care, care delivered at the community level, and demand-side interventions are also important, the focus of this article is on delivery and postnatal care at health facilities.

## EmNC Signal Functions Used in the Literature

In the absence of EmNC signal functions defined by UN organisations, we considered that researchers or policy makers may have developed their own sets of functions. We systematically reviewed the literature and identified eight documents listing EmNC signal functions (see Text S1): seven policy documents [10]–[16] and one peer-reviewed research article that used the Delphi method to develop quality of paediatric inpatient-care indicators, including some newborn care indicators [8]. 32 different functions were abstracted from the eight documents (see Table S1 in Text S1). There was widespread agreement for a few emergency functions (e.g., resuscitation and parenteral antibiotics), but most functions only appeared in one or two documents. Categories were inconsistent, with non-emergency routine care functions (e.g., thermal care and breastfeeding) included as emergency functions and some comprehensive functions, such as blood transfusion, suggested as basic functions.

## Proposed Signal Functions for Obstetric and Newborn Care

To develop a shortlist of proposed signal functions, the 32 functions abstracted from the literature review were considered together with key literature on newborn survival and prevention of stillbirths (including *The Lancet* neonatal survival series 2005, *International Journal of Gynecology and Obstetrics* intrapartum-related deaths series 2009, *BMC Pregnancy and Childbirth* preterm and stillbirth series 2010, *The Lancet* stillbirth series 2011, the Lives Saved Tool, and publications by the World Health Organization and the Partnership for Maternal, Newborn and Child Health). Criteria were that functions addressed the major causes of neonatal mortality—complications of preterm birth, intrapartum-related deaths, and severe infection—were highly effective interventions that were likely to be feasible in low-income settings and were easy to interpret and measure. We focused on delivery and postnatal care in health facilities, and excluded functions mainly delivered through antenatal or community care.

We then conducted an online survey among maternal and newborn health experts, contacting over 100 experts directly by email and placing the request on the Healthy Newborn Network blog. We asked the experts to choose signal functions from a list, to provide reasons for their choice, and give general feedback. 39 international experts, reporting an average of 16 years work experience in maternal health and 14 years in newborn health in low-income countries, responded. The functions with highest approval in the survey (see Text S2) and that accorded with the best evidence for mortality reduction in the literature and most persuasive and coherent rationale for feasibility in low-income countries according to the experts' free text comments were chosen for the final shortlist.

Table 1 presents the functions we suggest as routine and emergency obstetric and newborn care signal functions. Established functions (from the 2009 UN handbook [4]) are in italics, while new proposed functions are in normal print.

**Table 1:** Proposed obstetric and newborn signal functions.

Dimensions of Facility Care	Obstetric	Newborn
**General requirements** for health facility		
	Service availability 24/7	
	Skilled providers in sufficient numbers	
	Referral service to higher-level care, communication tools	
	Reliable electricity and water supply, heating in cold climates, clean toilets	
**A. Routine care** (for all mothers and babies)		
	Monitoring and management of labour using partograph	Thermal protection^a^
	Infection prevention measures (hand-washing, gloves)	Immediate and exclusive breastfeeding
	Active management of third stage of labour (AMTSL)^b^	Infection prevention including hygienic cord care^c^
**B. Basic emergency care** (for mothers and babies with complications)		
	*Parenteral magnesium sulfate for (pre-)eclampsia*	Antibiotics for preterm or prolonged PROM to prevent infection
	*Assisted vaginal delivery*	Corticosteroids in preterm labour
	*Parenteral antibiotics for maternal infection*	*Resuscitation with bag and mask of non-breathing baby*
	*Parenteral oxytocic drugs for haemorrhage*	KMC for premature/very small babies
	*Manual removal of placenta for retained placenta*	Alternative feeding^d^ if baby unable to breastfeed
	*Removal of retained products of conception*	Injectable antibiotics for neonatal sepsis
		(PMTCT if HIV-positive mother)^e^
**C. Comprehensive emergency care** (functions in addition to Basic)		
	*Surgery (e.g., C-section) including anaesthesia*	Intravenous fluids
	*Blood transfusion*	Safe administration of oxygen

Existing EmONC functions (from UN handbook) in italics.

a Thermal protection: drying baby immediately after birth, skin-to-skin with mother, wrapping, no bath in first 6 hours.

b AMTSL: oxytocin injection in thigh within 1 minute of delivery of baby, controlled cord traction, uterine massage after delivery of the placenta.

c Hygienic cord care: cutting with sterile blade, application of 4% chlorhexidine on tip of the cord and stump and no application of harmful substances (or clean and dry care in settings with low neonatal mortality and infection risk).

d Breastmilk expression and cup/spoon feeding.

e PMTCT: in brackets as not strictly a “newborn” function, but included for continuum of care; situational depending on HIV prevalence.

KMC, kangaroo mother care; PMTCT, prevention of mother to child transmission; PROM, premature rupture of membranes; 24/7, 24 hours a day 7 days a week.

The nature of “signal” functions is that they need not encompass all functions that are important for improving maternal and newborn health, but rather are a selection that “indicates” a certain level of care. While we based our selection on evidence and expert opinion, it remains arbitrary to some extent. For comprehensive EmNC in particular, no clear priorities emerged from the survey, with five functions receiving similar levels of support. We received yet further (and different) recommendations from our reviewers. As we felt it was desirable to limit the number of functions (to facilitate data collection, and to ensure that the criteria for success were not too onerous), we did not include availability of an incubator/radiant warmer, gastric tube feeding, or phototherapy despite their potential importance. Prevention of mother to child transmission (PMTCT) is a crucial function in settings with high HIV prevalence, but strictly speaking it is not a “newborn” function: while delivered at birth, it reduces infant and child rather than neonatal mortality. Nevertheless, considering the continuum of care and given PMTCT's situational importance and the fact that information on its provision is already widely collected, we included it in brackets. We noted with interest that in our online survey (see Text S2), more respondents approved of “intravenous fluids for shock” as an EmOC signal function than of “assisted vaginal delivery” (an existing signal function).

We also propose a set of general requirements important for facility functioning for obstetric and newborn care, including staffing, opening hours, reliable electricity, and water supply, as well as communication capability and referral services for lower-level facilities. Communication and referral are often neglected and many lives could be saved if patients were sufficiently stabilised and treatment was started before transport and if the referral facility was informed of the patient's arrival [17].

For some of the suggested Basic EmNC functions, it may be sufficient if Basic EmNC facilities (i.e., usually health centres) provide these to a limited degree, while comprehensive EmNC facilities (i.e., usually hospitals) provide them fully. For example, health centres might only be expected to start therapy for newborn sepsis with an intramuscular loading dose of antibiotics before referring to a hospital, or start and follow-up Kangaroo Mother Care (KMC) and breastmilk expression and cup-feeding for very low birthweight babies before and after hospital care.

## Signal Functions Collected with Available Facility Survey Tools

To assess which of our proposed functions were currently already collected at scale in more than one country, we examined facility survey tools from the International Health Facility Assessment Network (http://www.ihfan.org). Seven relevant tools [18]–[24], listed in Box 1, were identified. Five of these measure the established EmOC signal functions, but only two collect information on the provision of routine obstetric functions (Table 2). For instance, questions on routine use of the partograph are included in the Averting Maternal Death and Disability (AMDD) and Service Provision Assessment (SPA) tools, while the Rapid Health Facility Assessment (R-HFA) and the Service Availability and Readiness Assessment (SARA) tools check whether blank partographs are available, and the Health Facility Census (HFC), Service Availability Mapping (SAM), and Facility Audit of Service Quality (FASQ) do not enquire about any aspect of this function. Newborn care functions were infrequently included (Table 2), apart from newborn resuscitation and PMTCT. The recently updated SPA tool now enquires about provision of KMC for low birthweight babies and includes corticosteroids in preterm labour as a signal function. The AMDD tool measures provider knowledge of KMC and both AMDD and SARA measure availability of corticosteroids; none of the four other tools currently measure provision of these functions.

**Table 2:** Collection of general, obstetric, and newborn functions in large-scale facility assessments.

Dimensions of facility care	AMDD	HFC	SPA	FASQ	R-HFA	SAM	SARA
**General requirements**							
Service availability 24/7	**x**	**x**	**x**	x	**x**	—	(x)^a^
Number of skilled providers	**x**	**x**	**x**	**x**	**x**	**x**	**x**
Communication tools	**x**	**x**	**x**	**x**	**x**	**x**	**x**
Referral service to higher-level care	**x**	**x**	**x**	**x**	**x**	—	**x**
Electricity	**x**	**x**	**x**	**x**	**x**	—	**x**
Toilet or latrine	**x**	**x**	**x**	—	**x**	—	**x**
Water supply	**x**	**x**	**x**	**x**	**x**	**x**	**x**
**Routine obstetric care**							
Monitoring and management of labour with partograph	**x**	—	**x**	—	(x)^b^	—	(x)^b^
Infection prevention measures	(x)^b^	—	**x**	(x)^b^^,^ c	(x)^b^^,^ c	(x)^c^	(x)^b^
Active management of third stage of labour	**x**	—	(x)^c^^,^ d	(x)^d^	(x)^c^^,^ d	(x)^d^	(x)^d^
**Basic EmOC**							
*Parenteral magnesium sulfate for (pre-)eclampsia*	***x***	***x***	***x***	***x*** e	—	*(x)* d	***x***
*Assisted vaginal delivery*	***x***	***x***	***x***	***x***	*(x)* b	—	***x***
*Parenteral antibiotics for maternal infection*	***x***	***x***	***x***	***x*** e	—	*(x)* d	***x***
*Parenteral oxytocic drugs for haemorrhage*	***x***	***x***	***x***	***x*** e	*(x)* d	*(x)* d	***x***
*Manual removal of placenta for retained placenta*	***x***	***x***	***x***	***x***	—	—	***x***
*Removal of retained products of conception*	***x***	***x***	***x***	***x***	—	—	***x***
**Comprehensive EmOC**							
*Surgery (e.g., C-section)*	***x***	***x***	***x***	***x***	—	***x***	***x***
*Blood transfusion*	***x***	***x***	***x***	***x***	—	***x***	***x***
**Routine newborn care**							
Thermal protection	**x** f	—	**x**	—	—	—	—
Immediate and exclusive breastfeeding	**x** f	—	**x**	—	—	—	—
Hygienic cord care	**x** f	—	(x)^c^^,^ d	—	—	—	—
**Basic EmNC**							
Antibiotics to mother if preterm or prolonged PROM	—	—	—	—	—	—	—
Corticosteroids in preterm labour	(x)^d^	—	**x**	—	—	—	(x)^d^
*Resuscitation with bag and mask of non-breathing baby*	***x***	***x***	***x***	***x***	*(x)* b	—	***x***
KMC for premature/very small babies	**x** f	—	**x**	—	—	—	—
Alternative feeding if baby unable to breastfeed	(x)^b^	—	(x)^b^	—	—	—	—
Injectable antibiotics for neonatal sepsis	(x)^d^	(x)^g^	(x)^c^^,^ d	—	(x)^h^	(x)^i^	(x)^d^
(PMTCT if HIV-positive mother)	**x**	**x**	**x**	**x**	(x)^d^	**x**	**x**
**Comprehensive EmNC**							
Intravenous fluids	(x)^d^	(x)^g^	(x)^d^	—	—	—	(x)^d^
Safe administration of oxygen	(x)^b^^,^ f ^,^ j	(x)^b^	(x)^b^	—	—	(x)^b^	(x)^b^

Existing EmONC functions (from UN handbook) in italics.

a Only asked whether staff able to conduct C-section and anaesthesia are available 24/7.

b Only asked whether equipment available.

c Only asked whether staff received training.

d Only asked whether relevant drugs available.

e Asked about treatment for (pre-)eclampsia, for sepsis and for postpartum haemorrhage (not specifically what is done).

f Asked in the context of checking provider knowledge, given as answer option in a relevant question.

g Only asked about case management for severe pneumonia and severe dehydration for children in general (not specific to newborns).

h Only asked whether “antibiotics for newborn infections (except eye)” were available.

i Only asked about two injectable antibiotics (co-trimoxazole and ceftriaxon) that are not first choice for neonates due to risk of jaundice.

j Only asked whether special or intensive care provided.

FASQ, MEASURE Evaluation's Facility Audit of Service Quality; HFC, Health Facility Census by the Japan International Cooperation Agency; R-HFA, Rapid Health Facility Assessment of the Child Survival Technical Support Project; SAM, Service Availability Mapping of the World Health Organization; SARA, Service Availability and Readiness Assessment of the World Health Organization; SPA, Service Provision Assessment by MEASURE DHS.

**Box 1. Existing Facility-Survey Tools**

- Averting Maternal Death and Disability (AMDD): EmONC Needs Assessments
- Japan International Cooperation Agency (JICA): Health Facility Census (HFC)
- MEASURE DHS: Service Provision Assessment (SPA)
- MEASURE Evaluation: Facility Audit of Service Quality (FASQ)
- Child Survival Technical Support Project (CSTS+): Rapid Health Facility Assessment (R-HFA)
- World Health Organization (WHO): Service Availability Mapping (SAM)
- World Health Organization (WHO): Service Availability And Readiness Assessment (SARA).

Having identified the signal function areas captured with facility survey tools, another important facet is the way in which functionality is established, ranging from fairly simple to sophisticated. The simplest approach is to ask facility staff whether a function can be performed at their facility. Adding requirements for certain staff cadres and numbers, and drugs and equipment needed to perform the function, can improve the validity of the question [25]. This can be done either crudely by asking for the availability of a few tracer items, or in great detail by checking the actual presence of all necessary items in the facility as well as evaluating provider knowledge. Another option is to ask and/or verify whether a function has actually been provided at least once in a facility in the previous months, as suggested in the UN handbook on Monitoring EmOC [4].

A further sophistication involves establishing whether a function was provided when needed. For routine functions, which all mothers and babies should receive, and for which the denominator consists of all deliveries, it is feasible—where reliable records exist—to determine the proportion of deliveries in which a function is undertaken, e.g., proportion of deliveries in which a partograph was used. For emergency functions, both numerator and denominator need to be ascertained, which is more challenging, e.g., one would need to know not only how many newborns received injectable antibiotics, but also how many needed antibiotics (e.g., suffered from sepsis or pneumonia). Ntoburi and colleagues [8] used such an approach with paediatric quality of care indicators, including neonatal indicators.

## Recommendations for Facility Assessment of Signal Functions

We used literature review and expert opinion to identify and propose 23 signal functions for maternal and newborn emergency and routine care. This number appears to be the minimum needed to capture at least some of the important dimensions of routine and emergency care for mother and newborn. Limiting the number of functions helps ensure that these can be collected as part of a multi-purpose facility assessment without overburdening interviewers.

Our review of seven existing tools indicated that the majority of these functions are generally not collected, although the AMDD tool and the new SPA questionnaire have already integrated many relevant questions. We also noted that the existing tools used different approaches to assessing functionality, ranging from simple to sophisticated and data intensive.

Having an agreed list of functions makes it relatively easy to argue for the respective questions to be added to all existing tools. The issue of assessing functionality remains. While the more sophisticated methods clearly go further in capturing quality of care than the simpler ones, there is a trade-off with time and resources needed to assessing a given number of facilities. The simpler methods (e.g., asking about provision only or checking a few tracer items in addition to provision) can be adequate for assessing within-country variability, and they more easily allow for a full facility census to be conducted in large geographic areas. Multi-purpose facility censuses that include services beyond maternal and newborn health care may be attractive to, and more cost-effective for some countries, and by necessity will have fewer questions to devote to maternal and newborn functioning than single-purpose studies. In other settings, where records are available and most services provided, the next step might be to invest in capturing a wider range of functions and checking whether functions are really provided to all those in need. We recommend collecting information on health professionals and their tasks as well as on birth load for each facility in all surveys.

Secondly, although we used a variety of approaches to arrive at a set of signal functions, we recognize a limited evidence base is available. Ideally we would propose functions only after a good deal more formative research, however, the field is fast-moving, with new tools being developed and new data being collected. For this reason, we feel our review and suggestions are timely, though we propose that a research agenda is specified in parallel with adoption of our suggestions (see Box 2).

**Box 2. Recommendations for Data Collection and Research**

- Add proposed signal functions (at a minimum) to all future multi-country tools and country facility assessments
- Ensure data are collected on facility birth load and staffing
- Consider adding a broader range of signal functions to allow exploration of alternatives
- Validate existing functions by looking at their frequency in well-run facilities, in particular to come to an expert agreement on which EmNC functions should be provided at comprehensive level
- Define an agreed shortlist of tracer items (i.e., key drugs and equipment needed to perform a function) to verify function performance, derived from the item lists used by AMDD and others in various health facility assessments, e.g., in Kenya [26]
- Compare simple functional assessments (asking about provision with and without required staff, drugs and equipment tracer items) with more sophisticated assessments (verifying if functions done in last three months)
- Set and test benchmarks for routine and EmNC provision per number of births, and evaluate face validity of the indicator, including how it relates to known outcomes such as the neonatal mortality rate
- Create easy-to-use geographical interfaces integrated with Health Management Information Systems (HMIS) to facilitate continuous updating of information and monitoring of EmONC access

Ultimately, we feel we are in a strong position to recommend that the 23 routine and emergency care functions begin to be collected by countries. Once collected, they can be used in a variety of ways (see Box 3) to produce policy and programme relevant evidence to accelerate progress towards Millennium Development Goals 4 and 5.

**Box 3. How Our Proposed Signal Functions Can Be Used**

Countries that have already done health facility assessments that include all (or many) of the proposed signal functions (including those with new SPA and AMDD datasets) can use our suggestions to:

- Make preliminary national assessments of the ability of health facilities to provide routine and emergency obstetric and newborn care.
  1. Number and percentage that are CEmONC, BEmONC, provide routine care etc.
  2. Score of average number of functions for more nuanced assessment
- Make sub-national assessments at regional and even district level; use these data to target underserved areas
- Identify and plan around key bottlenecks at national level (e.g., are the main problems lack of qualified staff, a particular procedure, stock-outs of a particular drug?)
- Target specific facilities in specific areas for upgrading and strengthening
- Link facility data to data on population distribution and fertility rates (e.g., from a population census) to calculate service coverage by geographic area and for service planning [6]
- Link facility data to population data (e.g., from household surveys) in a geographic information system (possible if both datasets have GPS coordinates) to understand how access to services and their quality influence uptake [27]

## Conclusions

This article contributes a proposed set of signal functions for both mothers and babies, and for both emergency and routine care. We took into account evidence for interventions in the wider literature and the context in low-income countries and expert opinion. While this may not be the definitive set of signal functions, collecting the relevant data and promoting its use in a variety of ways will contribute to improving the quality of maternal and newborn care in low- and middle-income countries, helping to meet Millenium Development Goals 4 and 5. In a few years time, when sufficient experience has been gained in different settings, it may be appropriate to revisit both EmOC and EmNC and routine functions and provide more authoritative guidance and benchmarks.

## Supporting Information

Text S1 **Literature review findings on emergency newborn care functions.**
(PDF)

Text S2 **EmONC and routine care signal functions: expert opinion survey.**
(PDF)

## Abbreviations

AMDD: Averting Maternal Death and Disability
AMTSL: active management of the third stage of labour
EmNC: emergency newborn care
EmOC: emergency obstetric care
KMC: Kangaroo Mother Care
PMTCT: prevention of mother to child transmission
PROM: premature rupture of membranes
SPA: Service Provision Assessment

## References

[1] UN (2003) Indicators for monitoring the millennium development goals – definitions, rationale, concepts and sources. New York. Available: http://mdgs.un.org/unsd/mdg/Host.aspx?Content=Indicators/Handbook.htm. Accessed 12 October 2012.

[2] Graham WJ, Bell JS, Bullough CHW (2001) Can skilled attendance at delivery reduce maternal mortality in developing countries ? De Brouwere V, Van Lerberghe W, editors. Safe motherhood strategies: a review of the evidence. Antwerp: Studies in Health Services Organisation and Policy, 17, pp. 97–129.

[3] UNICEF WHO, UNFPA (1997) Guidelines for monitoring the availability and use of obstetric services. New York: United Nations Children Fund. Available: http://www.amddprogram.org/d/content/technical-resources. Accessed 12 October 2012.

[4] WHO UNFPA, UNICEF AMDD(2009) Monitoring emergency obstetric care, a handbook. Geneva: WHO. Available: http://www.who.int/reproductivehealth/publications/monitoring/9789241547734/en/index.html. Accessed 12 October 2012.

[5] Gabrysch S, Zanger P, Campbell OM (2012) Emergency obstetric care availability: a critical assessment of the current indicator. Trop Med Int Health 17: 2–8. 21831117 10.1111/j.1365-3156.2011.02851.x

[6] Gabrysch S, Zanger P, Seneviratne HR, Mbewe R, Campbell OMR (2011) Tracking progress towards safe motherhood: meeting the benchmark yet missing the goal? An appeal for better use of health-system output indicators with evidence from Zambia and Sri Lanka. Trop Med Int Health 16: 627–639. 21320245 10.1111/j.1365-3156.2011.02741.x

[7] WHO (1991) Essential elements of obstetric care at first referral level. Geneva: WHO. Available: http://helid.digicollection.org/en/d/Jh0227e/1.html. Accessed 12 October 2012.

[8] Ntoburi S, Hutchings A, Sanderson C, Carpenter J, Weber M, et al (2010) Development of paediatric quality of inpatient care indicators for low-income countries - A Delphi study. BMC Pediatr 10: 90. 21144065 10.1186/1471-2431-10-90 PMC3022793

[9] WHO (2005) The World Health Report 2005: make every mother and child count. Geneva: WHO. Available: http://www.who.int/whr/2005/en/index.html. Accessed 12 October 2012.

[10] Ministry of Health & Child Welfare Zimbabwe (2007) The Zimbabwe National Maternal and Neonatal Health Roadmap 2007–2015. Harare: Ministry of Health & Child Welfare Zimbabwe. 14–15 p. Available: http://www.who.int/pmnch/countries/zimbabwe_roadmap_web.pdf. Accessed 20 November 2011.

[11] Ministry of Health and Sanitation Sierra Leone (2008) Nationwide needs assessment for emergency obstetric and newborn care services in Sierra Leone. Freetown: Ministry of Health and Sanitation Sierra Leone. 4, 22–24, 74 p. Available: http://www.unicef.org/wcaro/wcaro_SL_EmNOC_assessment08.pdf. Accessed 20 November 2011.

[12] Ministry of Health Pakistan National Maternal, Newborn and Child Health Program. Definition of emergency obstetric and newborn care services. Available: http://www.mnch.gov.pk/eoc.php. Accessed 20 November 2011.

[13] Norway-Pakistan Partnership Initiative (NPPI) (2009) Feasibility study on result-based financial mechanisms for MNCH. 113–114 p. Available: http://www.researchcollective.org/Documents/NPPI%20FFS.pdf. Accessed 20 November 2011.

[14] Pakistan Initiative for Mothers and Newborns (PAIMAN) (2005) Health Facility Assessment (HFA). 15 p. Available: http://paiman.jsi.com/Resources/Docs/health-facility-assessment-paiman-original-districts.pdf. Accessed 20 November 2011.

[15] Republic of the Philippines - Department of Health (2008) Administrative order number 2008–0029. Implementing health reforms for rapid reduction of maternal and neonatal mortality. 3–4 p. Available: http://www.doh.gov.ph/files/ao2008-0029.pdf Accessed 29 March 2011.

[16] UNICEF (2007) Strategies and programming for newborn and child health, a UNICEF perspective, presentation by Nancy Terreri. 13–15 p. Available: http://www.esdproj.org/site/DocServer/NICP_Nancy_Terreri.pdf?docID=1044. Accessed 20 November 2011.

[17] Hussein J, Kanguru L, Astin M, Munjanja S (2011) What kinds of policy and programme interventions contribute to reductions in maternal mortality? The effectiveness of primary level referral systems for emergency maternal care in developing countries. Systematic review. London: EPPI-Centre, Social Sciences Research Unit, Institute of Education, University of London. Available: http://www.dfid.gov.uk/R4D/PDF/Outputs/SystematicReviews/Maternal-mortality-2011Hussein.pdf. Accessed 12 October 2012.

[18] AMDD Program AMDD Needs Assessment Toolkit. Available: http://amddprogram.org/d/content/needs-assessments. Accessed 20 November 2011.

[19] Health Facility Assessment Technical Working Group (2005) Profiles of health facility assessment methods. MEASURE Evaluation, USAID. Available: http://www.cpc.unc.edu/measure/publications/tr-06-36. Accessed 12 October 2012.

[20] MEASURE DHS Service Provision Assessment (SPA). Available: http://www.measuredhs.com/What-We-Do/Survey-Types/SPA.cfm. Accessed 10 August 2012.

[21] MEASURE Evaluation IHFAN (International Health Facility Asessment Network). Available: http://www.ihfan.org. Accessed 20 November 2011.

[22] MEASURE Evaluation Compendium of Maternal and Newborn Health Tools. Available: http://www.cpc.unc.edu/measure/publications/ms-02-09. Accessed 12 October 2012.

[23] WHO Service Availability Mapping (SAM). Available: http://www.who.int/healthinfo/systems/samquestionnaires/en/index.html. Accessed 20 November 2011.

[24] WHO Service Availability and Readiness Assessment (SARA). Available: http://www.who.int/healthinfo/systems/sara_related_links/en/index.html. Accessed 12 October 2012.

[25] Gabrysch S, Simushi V, Campbell OM (2011) Availability and distribution of, and geographic access to emergency obstetric care in Zambia. Int J Gynaecol Obstet 114: 174–179. 21669427 10.1016/j.ijgo.2011.05.007

[26] Opondo C, Ntoburi S, Wagai J, Wafula J, Wasunna A, et al (2009) Are hospitals prepared to support newborn survival? - An evaluation of eight first-referral level hospitals in Kenya. Trop Med Int Health 14: 1165–1172. 19695001 10.1111/j.1365-3156.2009.02358.x PMC2751740

[27] Gabrysch S, Cousens S, Cox J, Campbell OM (2011) The influence of distance and level of care on delivery place in rural Zambia: a study of linked national data in a geographic information system. PLoS Med 8: e1000394 doi:10.1371/journal.pmed.1000394. 21283606 10.1371/journal.pmed.1000394 PMC3026699

