# Supplementary material for: New Signal Functions to Measure the Ability of Health Facilities to Provide Routine and Emergency Newborn Care
Source: PLoS Med. 2012 Nov 13;9(11):e1001340. doi: 10.1371/journal.pmed.1001340 (PMC3496666; doi:10.1371/journal.pmed.1001340)
Supplement: Text S1 — Literature review findings on emergency newborn care functions. (PDF) [file pmed.1001340.s001.pdf]

Supplementary Information 1

Literature review findings on emergency newborn care functions

To identify documents listing signal functions for emergency newborn care (EmNC), we conducted a systematic literature review. Articles were included if they:

1. were about the functioning of FACILITIES, i.e. preparedness or readiness to deliver a certain level of facility care (and not about recommended interventions, packages, or community-level interventions alone),
2. recommended a SHORTLIST of functions or capabilities, not a very long list of all necessary items such as drugs, supplies, equipment pieces etc (although data on these items may be used to verify functions are ready to be performed), and
3. included at least three NEONATAL functions, not just one or two as part of a more general quality of care assessment.

Literature published since 2000 in Pubmed, Embase and Scopus databases was searched in August 2011 by combining MeSH terms for neonates and quality of care (e.g. “Infant, Newborn”, “Fetal Death”, “Paediatrics”, “Perinatal care, Standards”, “Quality of health care”, “Quality indicators, Health care”, “Clinical competence”) and using free text terms such as “Emergency neonatal care indicator” or “Newborn emergency signal functions”. A total of 4884 articles with a focus on our area of interest were located; all titles were screened, 113 abstracts were assessed and 17 articles were reviewed completely. Reference lists from retrieved articles and key articles in the field were also screened. Other published literature was searched using Intute (www.intute.ac.uk), Eldis (www.eldis.org) and Google (www.google.com) and free text terms relating to neonatal, emergency and signal functions or indicators. Eight documents (one research article and seven other published documents) fulfilled the inclusion criteria.

The emergency newborn care signal functions identified in these eight documents are listed below (direct quotes in italics) and summarised in Table S1.

Note: BEmOC and CEmOC functions are only mentioned when they differ from the UN guidelines.

1) Health Facility Assessment (HFA) 2005 – USAID, PAIMAN (page 15)
http://paiman.jsi.com/Resources/Docs/health-facility-assessment-paiman-original-districts.pdf

Basic EmNC
A facility that had each of following functions at the time of survey was considered as provider of the basic EmNC.

i. Basic newborn resuscitation.
ii. Warmth (drying and skin-to-skin contact).
iii. Eye prophylaxis (tetracycline eye ointment).
iv. Clean cord care.
v. Early and exclusive breast feeding.

Health facilities providing services both round the clock or day time was taken as providing the services.

Comprehensive EmNC
A facility that provided the following functions in addition to the function defined under basic EmNC was taken to be providing comprehensive EmNC:

i. Incubator available
ii. Advanced resuscitation support available e.g. intubation and respirator facility
iii. Pediatrics nursery available

2) Zimbabwe Maternal and Neonatal Health Roadmap 2007 (page 14+15)
http://www.who.int/pmnch/countries/zimbabwe_roadmap_web.pdf

Basic Emergency Obstetric and Neonatal [4] Care
***BEmONC** refers to an (abbreviated) list of services that can save the lives of women and newborns with obstetric and neonatal complications. A health facility qualifies as a BEmONC facility if it has performed each of the following "signal functions" at least once over the preceding 3 months:*

[4]According to the Zimbabwe Reproductive Health Steering Committee, the signal functions for emergency neonatal care are as outlined.

Basic Emergency Neonatal Care

- *Suctioning of newborns' airways*
- *Ventilating of newborns using Bag and mask*
- *Provision of thermal care*
- *Provision of parenteral antibiotics*
- *Provision of parenteral Vitamin K*
- *Provision of parenteral dextrose*

Comprehensive Emergency Obstetric and Neonatal Care
*To qualify as a **CEmONC** facility, all of the above services must be offered, but in addition the following functions are performed:*

Comprehensive Emergency Neonatal Care

- *Intubation and ventilation*
- *Narcain*
- *Surgery*

Though there are no clear guidelines on content of and coverage standards for EmNC, the MNH Road map has set out a package of interventions to form B/CEmNC.

3) Strategies and Programming for Newborn and Child Health, a UNICEF perspective. Presentation for USAID Scaling-up FP/MNCH Best Practices in Asia and Near East Technical Meeting Sept **2007**, by Nancy Terreri (Slides 13-15)
http://www.esdproj.org/site/DocServer/NICP_Nancy_Terreri.pdf?docID=1044

Existing health facilities should be able to:

- *Provide essential newborn care (drying, keep warm, breastfeeding, care of LBW etc)*
- *Provide care of the sick child (IMCI including newborn)*
- *Provide emergency newborn care (i.e. resuscitation)*
- *Identify and be able to treat newborn illnesses including giving antibiotics*
- *PMTCT+ if indicated*

IMCI + Newborn = Integrated Management of Newborn and Childhood Illness
IMNCI: Treats infants from birth to 5 years
All of IMCI plus: Birth to 2 month module to include:

- *Essential newborn care*
- *Emergency newborn care (i.e. resuscitation)*
- *Assessment of young infants for infection and diarrhoea*
- *Treatment and referral when required*
    - *Antibiotics (oral for pneumonia, oral plus injectable for very severe disease)*
    - *ORT*
- *Extra care (Kangaroo mother care) for LBW infants*
- *Support for initiation of early and exclusive breastfeeding and correction of problems*
- *Home care practices and danger sign awareness for the sick newborn*
- *Home visiting*

Basic EmONC
Everything from BEmOC plus:

- *Neonatal resuscitation with bag and mask*
- *Hypothermia Management (rewarming)*
- *Antibiotics for neonatal sepsis*
- *Essential newborn care*

Comprehensive EmONC
All of the above plus:

- *Assisted ventilation*

4) Administrative Order of the Department of Health in the Philippines, 2008: Implementing health reforms for rapid reduction of maternal and neonatal mortality (Pages 3+4 Definition of terms)
http://www.doh.gov.ph/files/ao2008-0029.pdf

***Basic Emergency Obstetric and Newborn Care (BEmONC) facilities** are capable of performing six signal obstetric functions, which include:*

(i) parenteral administration of oxytocin in the third stage of labor;
(ii) parenteral administration of loading dose of anticonvulsants;
(iii) parenteral administration of initial dose of antibiotics;
(iv) performance of assisted deliveries;
(v) removal of retained products of conception, and
(vi) manual removal of retained placenta.

BEmONC facilities are also capable of providing neonatal emergency interventions which include at the minimum:

- *(i) newborn resuscitation,*
- *(ii) treatment of neonatal sepsis/infection; and*
- *(iii) oxygen support.*

It shall also be capable of providing blood transfusion services on top of its standard functions.

***Comprehensive Emergency Obstetric and Newborn Care (CEmONC) facilities** can perform the six signal obstetric functions of a BEmONC and in addition, perform cesarean section and provide blood banking and transfusion services along with other highly specialized obstetric services.*
It is also capable of providing the following neonatal emergency interventions, which include at the minimum:

- *(i) newborn resuscitation,*
- *(ii) treatment of neonatal sepsis/infection,*
- *(iii) oxygen support for neonates, and*
- *(iv) management of low birth weight or premature newborn,*

along with other specialized neonatal services.

5) Nationwide Needs Assessment for Emergency Obstetric and Newborn Care Services in Sierra Leone. Ministry of Health and Sanitation **2008.** UNICEF, WHO and UNFPA assessment (page 4, page 13, page 22ff, page 74)

Similarly, substantial reductions in neonatal mortality require that newborns have access to the following services[15] ([15] Darmstadt Lancet series paper):

1. *Neonatal resuscitation with bag and mask*
2. *Hypothermia management (re-warming)*
3. *Antibiotics for neonatal sepsis (injectable and oral)*
4. *Essential newborn care*

Health facilities that performed the entire first six signal functions (refer to Section 1.2) are basic EmONC (BEmONC) facilities while those that performed all the eight signal functions are comprehensive EmONC (CEmONC) facilities. (i.e. it seems for BEmONC and CEmONC classification of facilities they used only the obstetric criteria, not the newborn ones, page 13)
There were other services that were considered vital to the outcome of pregnancies for both mothers and their unborn children. Health facility staff were asked if they had performed such services.

Figure 3.5: Proportion of CHCs and hospitals performing selected vital services

- *Administration of ARVs to newborns*
- *Administration of ARVs in maternity*
- *Rapid testing for HIV*
- *Intermittent presumptive treatment of malaria*
- *Early initiation of breast feeding*
- *LBW / Preterm care*
- *Parenteral antibiotics to the newborn*
- *Neonatal resuscitation*
- *Breech delivery*

There are no widely accepted signal functions for newborn care; nevertheless for the purpose of this assessment the provision of some essential newborn care or perinatal care services was investigated (fig 3.5).

6) Norway-Pakistan Partnership Initiative (NPPI): Feasibility study for results-based financial mechanisms for MNCH, commissioned by Sindh NMNCH Program and UNICEF, 2009. (Page 113, 114)
http://www.researchcollective.org/Documents/NPPI%20FFS.pdf

Table 11.1 Recommended Health Service Measures
Existing interventions / Potential interventions (including new strategies)
Areas:
Maternal survival and health
Causes of Deaths

- *Infection*
  - *Availability of antibiotics / Antibiotics for preterm rupture of membranes and suspected chorioamnionitis and post abortion care, Antenatal steroids in preterm labour*

Neonatal Health
Causes of Deaths

- *Asphyxia*
  - *Availability Cardio pulmonary resuscitation services & trained staff*
  - *Presence of Ambo bag Newborn Resuscitation*
  - *Post-asphyxial care including phenobarbitone*
- *Prematurity*
  - *Use of antenatal steroids*
  - *Presence of warmer/ heater*
  - *Presence of incubator*
  - *Availability of Oxygen supply*
- *Infection*
  - *Availability of nasal CPAP*
  - *Cutting cord with clean instrument (CDKs)*
  - *Breastfeeding initiated: In first hour, First day / Advice and support for early and exclusive breastfeeding, Breastfeeding education/promotion strategies*
  - *Provision of IV fluid administration services*

7) National Maternal, Newborn and Child Health Program, Ministry of Health, Govt of Pakistan. Definition of Emergency Obstetric and Newborn Care Services. Website last accessed 2011-10-25. http://www.mnch.gov.pk/eoc.php

Definition of Emergency Obstetric and Newborn Care Services:

*The provision of **Basic EmONC** services includes but is not limited to: intravenous and intra-muscular administration of drugs such as antibiotics, oxytocin and anticonvulsants; assisted vaginal delivery; manual removal of placenta; manual removal of retained products of an abortion or miscarriage; and stabilization and referral of obstetric emergencies not managed at the basic level. In terms of newborn emergencies, the required services at the basic EmONC level include management of neonatal infection, very low birth weight infants, complications of asphyxia and severe neonatal jaundice, (skills and supplies for intravenous fluid therapy, thermal care including radiant warmers, Kangaroo Mother Care, oxygen, parenteral antibiotics, intragastric feeding, oral feeding using alternative methods to breast feeding and breast feeding support.*

*The provision of **Comprehensive EmONC** services includes all of the services provided at the basic level, plus cesarean section, blood transfusion services, and newborn special care at the advanced level, such as intensive care neonatology units.*

8) Ntoburi et al: Development of paediatric quality of inpatient care indicators for low-income countires – A Delphi study, BMC Pediatrics **2010** http://www.biomedcentral.com/1471-2431/10/90

Top 5 indicators in the article (Table 3), the others from Additional file

Indicators

1. *The proportion of sick neonates with a diagnosis of neonatal sepsis prescribed the appropriate antibiotics (including correct choice of drug correct dose for weight and age frequency and route of administration according to guideline).*
2. *The proportion of babies born in hospital to HIV+ mothers who receive PMTCT therapy in line with national policy.*
3. *The proportion of babies born in hospital whose mothers have their HIV status known before delivery.*
4. *The proportion of babies aged <14 days prescribed routine Vitamin K in countries where this is national policy.*
5. *The proportion of newborn babies who get eye prophylaxis (Tetracycline Eye Ointment).*
6. *The proportion of sick neonates who are floppy or unable to breastfeed who have been correctly prescribed IV fluids or supplemental feeds according to post-natal age and weight (including correct feed type i.e. Expresses Breast Milk or formula milk ,dose and, route of administration).*
7. *The proportion of sick babies with a feeding chart appropriately filled.*
8. *A composite indicator of the proportion of neonates assessed completely. This includes: history of fever, difficulty in breathing, apnoeas, generalized convulsions, partial or focal fits, abnormal movement, difficulty in feeding , severe vomiting and, high pitched cry. In addition, the following key signs should be recorded: respiratory rate, temperature, central cyanosis, severe chest wall in drawing, grunting, capillary refill, pallor, skin pinch, floppy or unable to suck, bulging fontanel, jaundice, abdominal distension and, pus and inflammation of umbilicus severe skin pustules.*
9. *The proportion of babies who do not cry at birth who are given Bag Valve Mask ventilation. Note: aspects of the care of the mother in labour and of the immediate resuscitation of the newborn are beyond the scope of the current attempts to define indicators.*

Table S1: Emergency Newborn Care signal functions listed in the documents identified in the literature

		PAIMAN [1]	ZIMBABWE [2]	UNICEF [3]	PHILIPPINES [4]	SIERRA LEONE [5]	NPPI [6]	PAKISTAN* [7]	NTOBURI* [8]
Basic Emergency Newborn Care (BEmNC) functions	Routine ANC					IPT for malaria			
						Rapid HIV testing			Mother's HIV status known
	ANC for complications						Antenatal steroids in preterm labour		
							Antibiotics for preterm ROM & suspected chorioamnionitis		
	Routine delivery					Breech delivery			
		Warmth (drying & skin-to-skin contact)	Provision of thermal care	Drying, keep warm					
		Early & exclusive breast feeding (BF)		Support for initiation of early & exclusive BF		Early initiation of BF	Advice & support for early & exclusive BF	BF support	
		Clean cord care					Cutting cord with clean instrument		
		Eye prophylaxis (tetracycline ointment)							Eye prophylaxis (tetracycline ointment)
			Provision of parenteral Vit. K						Babies <14 days prescribed routine Vit.K
				Essential newborn care		Essential newborn care			
	Complicated delivery					Administration of ARVs in maternity & to newborns			PMTCT to mother in line with national policy
			Suctioning of newborns' airways						
		Basic newborn resuscitation	Ventilating of newborns using bag & mask	Neonatal resuscitation with bag & mask	Newborn resuscitation	Neonatal resuscitation with bag & mask	Newborn resuscitation, Presence of Ambo bag		Bag Valve Mask ventilation for non-crying baby at birth
									Complete assessment of sick newborn (composite of history & physical exam)
				Hypothermia management (rewarming)		Hypothermia management (rewarming)	Presence of warmer / heater	Thermal care including radiant warmers	

				Extra care (Kangaroo mother care) for LBW infants		LBW / Preterm care		Kangaroo Mother Care	
			Provision of parenteral antibiotics	Antibiotics for neonatal sepsis	Treatment of neonatal sepsis / infection	Antibiotics for neonatal sepsis (injectable & oral)		Management of neonatal infection, parenteral antibiotics	Appropriate antibiotics for neonatal sepsis
								Oral feeding using alternatives to breast feeding	Supplemental feeds (e.g. expressed breast milk or formula) if baby unable to breastfeed
								Intragastric feeding	
			Provision of parenteral dextrose				Provision of IV fluid administration services	IV fluid therapy	IV fluids
									Appropriately filled feeding chart for sick babies
		Services both round the clock or day time							
					Oxygen support		Availability of oxygen supply	Oxygen	
					Blood transfusion				
**Additional functions for Comprehensive Emergency Newborn Care (CEmNC)**					Management of LBW or premature				
		Incubator available					Presence of incubator		
		Advanced resuscitation support e.g. intubation & respirator facility	Intubation & ventilation	Assisted ventilation			Availability of nasal CPAP		
							Post-asphyxial care incl. phenobarbitone		
		Paediatrics nursery			Other specialized neonatal services			Newborn special care at advanced level, such as NICU	
			Narcain						
			Surgery						

BF = breastfeeding, IPT = intermittent preventive treatment, IV = intravenous, LBW = low birth weight, NICU = neonatal intensive care unit, ROM = rupture of membranes, Vit.K = vitamin K

* The definition of the Pakistan Ministry of Health and Ntoburi do not separate basic and comprehensive care, and Ntoburi gives indicators as percentage of cases with a certain condition that received the appropriate treatment; functions were thus rephrased (for Ntoburi) and positioned appropriately.

1. Pakistan Initiative for Mothers and Newborns (PAIMAN). *Health Facility Assessment (HFA)*. 2005. p. 15. http://paiman.jsi.com/Resources/Docs/health-facility-assessment-paiman-original-districts.pdf.
2. Ministry of Health & Child Welfare Zimbabwe. *The Zimbabwe National Maternal and Neonatal Health Roadmap 2007-2015*. 2007. Harare. p. 14-15. http://www.who.int/pmnch/countries/zimbabwe_roadmap_web.pdf.
3. UNICEF. *Strategies and Programming for Newborn and Child Health, a UNICEF perspective, presentation by Nancy Terreri*, in *USAID Scaling-up FP/MNCH Best Practices in Asia and Near East Technical Meeting*. 2007. p. 13-15. http://www.esdproj.org/site/DocServer/NICP_Nancy_Terreri.pdf?docID=1044.
4. Republic of the Philippines - Department of Health. *Administrative Order No. 2008-0029. Implementing health reforms for rapid reduction of maternal and neonatal mortality*. 2008. p. 3-4. http://www.doh.gov.ph/files/ao2008-0029.pdf
5. Ministry of Health and Sanitation Sierra Leone. *Nationwide Needs Assessment for Emergency Obstetric and Newborn Care Services in Sierra Leone.* 2008. Freetown. p. 4, 22-24, 74. http://www.unicef.org/wcaro/wcaro_SL_EmNOC_assessment08.pdf.
6. Norway-Pakistan Partnership Initiative (NPPI). *Feasibility study on result-based financial mechanisms for MNCH*. 2009. p. 113-114. http://www.researchcollective.org/Documents/NPPI%20FFS.pdf.
7. Ministry of Health Pakistan. *National Maternal, Newborn and Child Health Program. Definition of Emergency Obstetric and Newborn Care Services*. [cited 2011-11-20]; Available from: http://www.mnch.gov.pk/eoc.php.
8. Ntoburi S, Hutchings A, Sanderson C, Carpenter J, Weber M, and English M. *Development of paediatric quality of inpatient care indicators for low-income countries - A Delphi study.* BMC Pediatr, 2010. **10**: p. 90. Available at: http://www.biomedcentral.com/1471-2431/10/90
