# Supplementary material for: New Signal Functions to Measure the Ability of Health Facilities to Provide Routine and Emergency Newborn Care
Source: PLoS Med. 2012 Nov 13;9(11):e1001340. doi: 10.1371/journal.pmed.1001340 (PMC3496666; doi:10.1371/journal.pmed.1001340)
Supplement: Text S2 — EmONC and routine care signal functions: expert opinion survey. (PDF) [file pmed.1001340.s002.pdf]

Supplementary Information 2

EmONC and routine care signal functions: expert opinion survey

**Questions and functions**	Function count	Function percent	Function chosen
Which of the following would you choose as signal functions for **routine maternal / obstetric care** in health facilities? (routine = what all mothers should receive) Responses to question n=37; Additional comments n=17			
Blood pressure measurement at admission	34	91.9%	*See* *
Monitoring and management of labour using partograph	34	91.9%	X
Infection prevention measures (gloves, sterile instruments)	33	89.2%	X
Companion allowed during labour and delivery	25	67.6%	
Active management of third stage of labour (AMTSL)	35	94.6%	X
* *Blood pressure measurement was not chosen as a signal function because it was considered to be part of "monitoring and management using partograph" which was chosen.*			
Which of the following would you choose as signal functions for **routine newborn care** in health facilities? (routine = what all babies should receive) Responses to question n=36; Additional comments n=12			
Thermal protection (drying, skin-to-skin, delay of bath)	36	100.0%	X
Immediate (within 1 hour) and exclusive breastfeeding	35	97.2%	X
Hygienic cord care (chlorhexidine or clean dry care)	34	94.4%	X
Which of the following would you choose as signal functions for **basic emergency obstetric care** in health facilities? (Pick a maximum of 6 functions) Responses to question n=35; Additional comments n=14			
Parenteral anticonvulsants for (pre-)eclampsia	34	97.1%	X
Assisted vaginal delivery (forceps or vacuum extraction)	22	62.9%	X
Parenteral antibiotics for maternal infection	34	97.1%	X
Parenteral oxytocic drugs for haemorrhage	35	100.0%	X
Manual removal of placenta for retained placenta	30	85.7%	X
Removal of retained products of conception (e.g. MVA)	28	80.0%	X
Intravenous fluids for shock	30	85.7%	*See* *
* *We did not change the existing set of EmOC signal functions.*			

Which of the following would you choose as signal functions for **basic emergency newborn care** in health facilities? (Pick a maximum of 6 functions) Responses to question n=36; Additional comments n=14			
Antibiotics for preterm or prolonged PROM to prevent infection	22	61.1%	X
Corticosteroids in preterm labour	20	55.6%	X
Resuscitation with bag and mask of non-breathing baby	36	100.0%	X
Skin-to-skin / KMC for preterm and very small babies	30	83.3%	X
Alternative feeding if baby unable to breastfeed (breastmilk expression, cup/spoon)	16	44.4%	X
Injectable antibiotics for neonatal sepsis	31	86.1%	X
PMTCT if HIV-positive mother	27	75.0%	(x)
Elective induction of labour for post-term delivery (>41 weeks)	1	2.8%	
Postnatal parenteral Vitamin K supplementation for small babies	8	22.2%	
Delayed cord clamping for preterm babies	3	8.3%	
Which of the following would you choose as signal functions for **comprehensive emergency obstetric care** in health facilities? Responses to question n=36; Additional comments n= 9			
Surgery (e.g. C-section) including anaesthesia	36	100.0%	X
Blood transfusion	36	100.0%	X
Which of the following would you choose as signal functions for **comprehensive emergency newborn care** in health facilities? (Pick a maximum of 5 functions) Responses to question n=33; Additional comments n=12			
Nasogastric tube if baby not feeding	26	78.8%	*See **
Incubator or heated cot for very small babies	24	72.7%	
Administration of oxygen if low saturation	24	72.7%	X
Phototherapy for jaundice	23	69.7%	
Intravenous fluids for shock **	23	69.7%	X
Surfactant for respiratory distress syndrome in preterm babies	6	18.2%	
Continued positive airway pressure (CPAP) for respiratory distress syndrome	20	60.6%	
** Persuasive arguments were made against nasogastric tube on the grounds of rarity of indication and practice (only preterm babies below 1.2kg) in a hospital setting in Ghana.*<br>*** Some experts pointed out that i.v. fluids are not just important for shock, therefore omitted "for shock" in the final table*			
Further comments n=13			
